# Correlation between Blood Pressure and Skin Health in Korean College Female Students

**DOI:** 10.18502/ijph.v49i7.3594

**Published:** 2020-07

**Authors:** Jeong-Hyun LEE, Yeona KIM, Wi-Young SO

**Affiliations:** 1. Department of Beauty Art, Youngsan University, Busan, Republic of Korea; 2. Sports and Health Care Major, Korea National University of Transportation, Chungju-si, Republic of Korea

## Dear Editor-in-Chief

According to WHO report, 1,130 million people have hypertension worldwide, and less than 20% of people with hypertension have the problem under control. Further, 25% of men and 20% of women were reported to have hypertension in 2015 (1). Additionally, according to the Fifth Korea National Health and Nutrition Examination Survey in 2017, the prevalence of hypertension in men and women over 30 years was 32.3% and 21.3%, respectively. Thus, hypertension is becoming a social and public health problem in Korea (2).

Skin health is also one of the prevailing health conditions. Although many previous studies have investigated the association between blood pressure and public health problems (3–4), to the best of our knowledge, an association between blood pressure and skin health conditions has not been demonstrated. Therefore, this study aimed to examine the correlation between blood pressure and skin health outcomes in Korean college female students.

This study enrolled 59 Korean college female students on November 2019 at a Beauty & Healthcare Center of Youngsan University, Busan, Korea. Inclusion criteria were as follows: those who do not drink and smoke, those with no history of drug use, and those without regular exercise training. The participants provided informed consent and all study procedures were approved by the Institutional Review Board of Youngsan University, Busan, Korea (YSUIRB-201907-HR-054-02).

Blood pressure and resting heart rate were measured after 10 minutes of sitting while at rest using an autonomic blood pressure measuring device (BPBIO-320, Inbody, Seoul, Korea). For measuring skin health conditions, participants were asked to not use makeup prior to conducting the analysis. For the analysis, participants were instructed to wash the face with only water; the hair was tossed backward and secured using a turban after 10 minutes of natural drying. The skin condition was measured using the JANUS facial analyzing system (JANUS-2 system, Janus imaging system PSI Co. Ltd, Seoul, Korea). The facial analyzer can measure pores (%), wrinkles (%), pigmentation with ultraviolet light (%), pigmentation with polarizing light (%), sebum level (total count), porphyrin content (%), and skin tone (%). It can analyze multiple parameters of the whole face and extract the analyzed data using a 10 megapixel Digital Single-Lens Reflex Camera. All results are presented as mean ± standard deviation. Pearson-correlation analysis was performed to determine the association between blood pressure and skin health outcomes using SPSS version 18.0 (IBM Corp., Armonk, NY, USA). Statistical significance was set at *P*<0.05.

The characteristics of participants are shown in Table 1. The outcomes of the Pearson correlation analysis are shown in Table 2.

**Table 1:** Characteristics of participants (n=59)

*** Variables ***		*** Means ± standard deviations ***
Age (yr)		20.90 ± 1.49
Height (cm)		160.31 ± 5.14
Weight (kg)		60.17 ± 15.33
Body mass index (kg/m ^ 2 ^ )		23.37 ± 5.49
Cardiovascular function	Systolic blood pressure (mmHg)	112.58 ± 10.36
	Diastolic blood pressure (mmHg)	72.44 ± 9.90
	Resting heart rate (beats/min)	86.93 ± 15.24
Skin health outcomes	Pores (%)	49.08 ± 10.26
	Wrinkles (%)	19.14 ± 13.26
	Pigmentation with ultraviolet light (%)	11.81 ± 6.17
	Pigmentation with polarizing light (%)	11.31 ± 6.56
	Sebum level (total count)	95.51 ± 155.03
	Porphyrin content (%)	30.36 ± 24.56
	Skin tone (%)	56.14 ± 3.20

**Table 2:** Results of Pearson correlation analysis between cardiovascular function and skin health (N=59)

*** Variables ***	*** Systolic blood pressure (mmHg) ***		*** Diastolic blood pressure (mmHg) ***		*** Resting heart rate (beats/min) ***	
	*** r ***	*** P ***	*** r ***	*** P ***	*** r ***	*** P ***
Pores (%)	0.209	0.112	0.271	0.038^*^	−0.050	0.707
Wrinkles (%)	0.315	0.015^*^	0.263	0.045^*^	0.050	0.708
Pigmentation with ultraviolet light (%)	0.129	0.329	−0.015	0.910	−0.120	0.364
Pigmentation with polarizing light (%)	0.090	0.498	−0.087	0.513	−0.190	0.150
Sebum level (total count)	0.235	0.073	0.109	0.413	−0.020	0.879
Porphyrin content (%)	−0.047	0.723	−0.083	0.532	0.039	0.769
Skin tone (%)	−0.240	0.067	−0.109	0.413	−0.096	0.470

* *P* < 0.05; tested by Pearson correlation analysis

The analyses revealed that pores have a significant correlation with diastolic blood pressure (r=0.271, *P*=0.038). Further, wrinkles were shown to have a significant correlation with systolic blood pressure (r=0.315, *P*=0.015) and diastolic blood pressure (r=0.263, *P*=0.045). However, there were no correlations between pigmentation with ultraviolet light, pigmentation with polarizing light, sebum level, porphyrin content, and skin tone with systolic blood pressure, diastolic blood pressure, and resting heart rate.

In conclusion, pores and wrinkles are positively correlated with blood pressure. Future studies are required to examine the effect of blood pressure on pores and wrinkles.
